# Task’s Choice: Pruning-Based Feature Sharing (PBFS) for Multi-Task Learning

**DOI:** 10.3390/e24030432

**Published:** 2022-03-21

**Authors:** Ying Chen, Jiong Yu, Yutong Zhao, Jiaying Chen, Xusheng Du

**Affiliations:** 1 School of Software, Xinjiang University, Urumqi 830008, China; 2 College of Information Science and Engineering, Xinjiang University, Urumqi 830046, China

**Keywords:** multi-task learning, information sharing, deep learning, transfer learning

## Abstract

In most of the existing multi-task learning (MTL) models, multiple tasks’ public information is learned by sharing parameters across hidden layers, such as hard sharing, soft sharing, and hierarchical sharing. One promising approach is to introduce model pruning into information learning, such as sparse sharing, which is regarded as being outstanding in knowledge transferring. However, the above method performs inefficiently in conflict tasks, with inadequate learning of tasks’ private information, or through suffering from negative transferring. In this paper, we propose a multi-task learning model (Pruning-Based Feature Sharing, PBFS) that merges a soft parameter sharing structure with model pruning and adds a prunable shared network among different task-specific subnets. In this way, each task can select parameters in a shared subnet, according to its requirements. Experiments are conducted on three benchmark public datasets and one synthetic dataset; the impact of the different subnets’ sparsity and tasks’ correlations to the model performance is analyzed. Results show that the proposed model’s information sharing strategy is helpful to transfer learning and superior to the several comparison models.

## 1. Introduction

**Figure 1:**
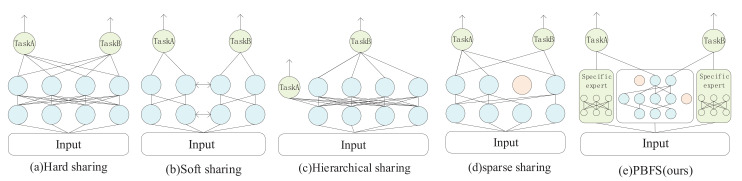
Most of the existing multi-task learning architecture. (**a**) Hard parameter sharing. (**b**) Soft parameter sharing. (**c**)Hierarchical sharing. (**d**) Sparse sharing. (**e**) The proposed model PBFS.

Multi-task learning aims to augment the generalization performance by sharing information among multiple tasks, which has succeeded in computer vision, recommendation systems, and natural language processing [1,2,3,4,5]. Researchers have conducted many experiments based on their model architecture to show the usefulness of multi-task learning. The existing information-sharing mechanisms in multi-task learning are mainly divided into four categories: hard parameter sharing, soft parameter sharing, hierarchical sharing, and sparse sharing. As the most basic information-sharing method, hard parameter sharing in Figure 1a, which was proposed by [5], appends a tower layer (a simple dense network to obtain the final output of the task) on the top of the identical hidden layer representation, so the same parameters are utilized by different tasks; many studies [6,7,8] based on this method have achieved excellent performance. Each task in the soft parameter sharing mechanism in Figure 1b has independent parameters and subnet, but their information can be mutually learned. This method usually improves the prediction accuracy [9] by making parameters as similar as possible with regularization or fusing information with a gated network and attention mechanism.

The hierarchical sharing [10,11] in Figure 1c considers the progressive relationship between tasks, which means that task A may be a subtask of another one, thus placing them on different network layers. The sparse sharing mechanism [12] in Figure 1d is based on the idea of the lottery ticket hypothesis, which argues that in any dense base network, an optimal subnet could be found for each task to learn the required information. Since the same base network is used, different tasks will automatically select the information through IMP (iterative magnitude pruning).

However, the above methods have certain limitations. When tasks conflict, hard parameter sharing is easily affected by negative transferring, which is harmful to the model performance [9]. Soft parameter sharing depends on a complex manual design model architecture, and the discovery of effective information-sharing strategies often requires researchers to pay a major expenditure of time and effort. The hierarchical sharing mechanism relies on tasks’ relations, and the deployment of tasks is a complex problem without prior knowledge [13]. Various tasks in sparse sharing rely on the same base network without considering the interaction and differences of tasks, and it is hard to learn in training [14]. Meanwhile, the seesaw phenomenon is also one of the significant difficulties that most multi-task models face, which means that improvements in one task often cause performance degradation of other tasks.

This paper proposes a multi-task learning model based on parameters pruning to address the above problems shown in Figure 1e, which combines a soft parameter sharing mechanism and model pruning. On an architecture that separates task-specific experts and shared experts, the parameters of the latter can be partially trimmed. Specifically, each task has a task-specific expert, and shared experts are connected by several tasks, which are the pruned base network, simultaneously. Each task selects the optimal shared subnet by conducting iterative quantitative pruning on the shared expert, and then the opted subnet is combined with task-specific experts for training. So in this model, tasks have their individual subnet, whether it is a task-specific or shared subnet. The advantages of this method are (a) low-relevance or conflicting tasks can be compatible in the same model without being mutually affecting, (b) no reliance on task characteristics to manually design a shared architecture, and (c) noise parameters that will decrease the performance of the model can be pruned.

Our paper contributions can be summarized as follows:

1. A soft sharing model jointed with parameter pruning is designed, in which each task has an independent subnet, and the pruning is used to learn the information of shared experts on the parameter level;
2. A task-specific pruning strategy is used to find the optimal subnet for each task so that it can automatically learn to select the hidden representations and cut off harmful noise;
3. Establish a shared pool based on the difference of the tasks’ relevance, and define the limits of whether diverse tasks can prune a base shared expert so that more than two tasks have an adaptive sharing scheme;
4. Experiments in three benchmark public datasets demonstrated that the proposed model achieves considerable improvement compared with single-task learning and several soft parameter sharing models. Meanwhile, we found that our pruning strategy can avoid the impact of the seesaw phenomenon, which appeared in many MTL models.

## 2. Related Work

### 2.1. Multi-Task Learning for Deep Learning

With the target of improving generalization and prediction by utilizing the task-specific information contained in the training signals of related tasks [5], researchers widely apply multi-task learning for optimizing multiple targets simultaneously. Based on deep learning, traditional MTL in DNNs can be divided into two methods: hard parameter sharing and soft parameter sharing.

Hard parameter sharing supposes that the latent feature of different tasks can be partially extracted to the same hidden representations, which are regarded as the maximum common knowledge among various tasks. There have been some studies applying this structure and idea for information learning. ESMM [1] applies two auxiliary tasks and shares partial embedding parameters among different tasks to improve the performance of the main task. Hadash G. et al. [2] proposed a progressive multi-task model to construct the correlations between tasks explicitly. Similarly, Qin Z. et al. [15] introduced LSTM as the sharing bottom layer into a hard parameter sharing multi-task model to learn common information. However, hard sharing is adversely susceptible to negative transfer with complicated tasks. Clarkson G. et al. [16] considered information asymmetry and proposed a novel knowledge-sharing method to address task conflicts. Meanwhile, cross-stitch network [3] and sluice network [17] both select the fuse representations of different tasks by learning linear combination weights. Unfortunately, the task’s latent feature picked by the same static weights between loosely related tasks will significantly reduce the model’s predictive ability.

To address the above issues, many researchers have argued that each task should have an independent subnet to alleviate the effect of noise parameters from other data samples. In this way, several works introduced a gated structure or attention network to improve the performance of each task subnet, such as MoE [18], to provide a new link between two different approaches and first apply gated-network to combine experts. MMOE [9] upgrades MoE with setting up a particular gated network for each task by considering sample independence. Jiejie Z. et al. [19] used multi-head self-attention to learn different subspaces at different feature sets. PLE [13] improved the traditional gated structure and removed unnecessary connections between experts to alleviate harmful parameter interference between common and task-specific knowledge.

In particular, hierarchical sharing approaches [10], a novel idea in MTL study, reason that low-level tasks are better kept at the lower layers, enabling the high-level tasks to use the shared representation of the lower-level tasks. Then researchers present a multi-task learning architecture with deep bi-directional RNNs, where different task supervision can happen at different layers.

### 2.2. Sparse Networks

Deep learning can automatically learn the features required by the target profiting from the rapid growth of neural networks. Nevertheless, extensive professional knowledge and experiments are expected to design a well-behaved neural network with high computational cost, limiting its application in many fields. Therefore, many techniques seeking more sparse networks have emerged, such as NAS [20], quantization [21,22], pruning [23,24,25], etc., to reduce computational complexity and risk of overfitting without harming accuracy.

Given a set of candidate neural network structures called the search space, in each iteration of the search process, NAS aims to search for a sub-network smaller than the original model, and then the network structure is gradually optimized until the optimal sub-structure is found [4,26,27]. Many multi-task models use NAS to find a more suitable sparse subnet for each task to improve the accuracy of multi-objective prediction. For example, in [28], binary variables are used to control the connection between subnets, and AutoML technology is applied to explore the best structure of the task.

However, the NAS technology training and verification process are very time consuming, and some time reducing methods will be unfavorable to the model. In contrast, pruning provides an easier and more efficient approach to reduce storage occupancy, communication bandwidth, and computational complexity [4]. Frankle J. et al. [29] argued that a randomly initialized, dense neural network always contains a more sparse network, which can match the test accuracy of the original network after being trained. Actually, this sub-network can train faster but attach higher test accuracy and generalize better. LT4REC [14] applied this hypothesis to recommendation systems, which can automatically and flexibly learn which neuron weights to be shared without artificial experience. Sun T et al. [12] systematically built sparse sharing architectures for multiple tasks by introducing the pruning technique, thereby proposing a novel parameter sharing mechanism, named sparse sharing, that can achieve consistent improvement while requiring fewer parameters.

## 3. Pruning-Based Feature Sharing Architecture

### 3.1. Base Model

**Figure 2:**
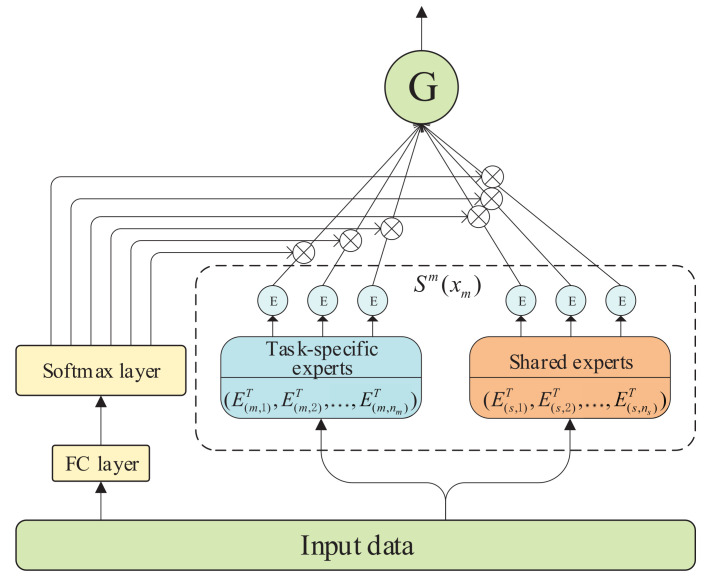
Structure of gated network.

Our base model is motivated by CGC [13]; in CGC [13], shared experts are detached with task-specific experts, but then both are combined through a gated network for selective fusion. Specifically, the gated network (shown in Figure 2) is designed to capture the differentiation and interaction between experts, which is based on a single-layer feed-forward network with softmax as the activation function, and input as the selector to calculate the weighted sum of the selected vectors. Similarly, a tower layer is laid out on the top of the gated network for receiving independent tasks’ target representation. The output of task *m* can be formally expressed as:

(1) $$\phi^{m}\left(x_{m}\right)=w^{m}\left(x_{m}\right)S^{m}\left(x_{m}\right)$$

where $x_{m}$ is the input of task *m*, $w^{m}$ represents the weighting function to calculate the weight vector of task *m* through linear transformation and softmax activation function, $S^{m}$ is a united matrix made up of all output vectors, including task *m*’s specific experts and shared experts, and their calculation formula is given as follows:

(2) $$w^{m}\left(x_{m}\right)=\mathrm{SoftMax}\left(w_{g}^{m}\left(x_{m}\right)\right)$$

(3) $$S^{m}\left(x_{m}\right)=\left[E_{\left(m,1\right)}^{T},E_{\left(m,2\right)}^{T},\ldots E_{\left(m,n_{m}\right)}^{T},E_{\left(s,1\right)}^{T},E_{\left(s,2\right)}^{T},\ldots E_{\left(s,n_{s}\right)}^{T}\right]$$

where $w_{g}^{m}\in R^{\left(n_{m}+n_{s}\right)\times d}$ is a trainable matrix, *d* expresses the dimension of the input data, $n_{m}$ and $n_{s}$ are the number of task *m*’s specific experts and shared experts, respectively, and $E_{\left(m,n_{m}\right)}^{T}$ and $E_{\left(s,n_{s}\right)}^{T}$ represent the transposes of different expert’s output. Finally, the prediction of task *m* is

(4) $$y_{x_{m}}^{m}=t^{m}\left(\phi g^{m}\left(x_{m}\right)\right)$$

where $t^{m}$ indicates the tower layer of task *m*.

Since then, a dense base network has been established, which removes connections between a task’s tower layer and the task-specific experts of other tasks and retains the traditional hard parameter sharing mechanism in which different tasks use the same shared expert representation.

### 3.2. Model Architecture

The deep learning based multi-task model optimizes the performance of each task by training in parallel, considering the differences and interaction between tasks. The proposed model comprises task-specific experts and shared pools to consider the differences and interactions between tasks. The former is a unique subnet for each task. The latter connects all tasks with special associations and learns common information through specific parameter pruning. In the following part, we formally define the main architecture of the proposed model.

Given *M* tasks to be learned, with each *m* process, a corresponding samples space $D_{m}\in \left(N_{m}\times d_{m}\right)$, where $N_{m}$ denotes the number of *m*’s sample, $d_{m}$ indicates data dimension, and the task’s correlation score is calculated with the Pearson coefficient, is defined as ${\mathrm{COOR}}_{M}$, which is a M*M symmetric matrix, with the diagonal elements being 1.

In traditional research [9,13], different tasks utilize identical shared experts. However, it will cause noise for tasks with large differences in relevance. Therefore, we define a shared pool *s* (s=1,2,…,S), with each s=<$Q\left[s\right],A_{s}$>, where Q[s](Q[s]⊆M) is a set of the task’s index, which is connected with shared pool *s*, and $\mathrm{error}(2⩽A_{s}⩽M)$ is the number of tasks that appears in Q[s]. Then, its shared mean factor $SMF_{s}$ is defined as follows:

(5) $$SMF_{s}=\left\{\begin{array}{c} {\mathrm{COOR}}_{M},A_{s}=2\\ \frac{\sum_{\left(i,j\right)\in Q_{\left[s\right]}}COOR_{M}^{<i,j>}}{C_{A_{s}}^{2}},A_{s}>2 \end{array}\right.$$

where *C* is the combination number formula in mathematics. When there are only two tasks in a shared pool, $SMF_{s}$ ($0<SMF_{s}<1$) is the correlation between the two tasks. Otherwise, for any associated task pair <i,j>(i≠j,i,j⊆M)for the new task*k*, the condition for sharing data in *s* is: $\left|SMF_{s}-{\mathrm{COOR}}_{M}^{<i,j>}\right|<\alpha$, and α is an adjustable empirical parameter that controls the selection of tasks in the shared pool *s*.

In this way, to task *m*, a related sharing pool set $U_{m}\left(U_{m}\subseteq S\right)$ can be obtained. Different prune strategies are applied to shared pools in the set, which we will cover in detail in the next section. Finally, the output of task m’s sharing pool can be mathematically formulated as follows:

(6) $$P_{m}=\sum_{s=0}^{U_{m}}f_{s}\left(\lambda ,\sigma \left(x_{s}\right)\right)$$

where *f* is the pruning function, λ denotes the pruning parameter of the task *m* for the shared pool *s*, namely the target sparseness, σ means the specified activation function, and $x_{s}$ is the input data of the shared pool *s*.

Each task also has the output of a task-specific subnet, whose input data are the corresponding samples related to the task, focusing on learning the information required by the corresponding task. In this paper, the specific task subnet we use is the MLP network, and the activation function depends on the task attributes. Therefore, the output of a specific task subnet for task *m* is

(7) $$O_{m}=\sigma \left(wx_{m}\right)$$

where σ denotes the activation function.

In summary, for task *m*, we not only learned the knowledge through information sharing with other tasks, but also obtained the specific knowledge of the task, then let it pass through a gated network with a selection function to obtain the output. Therefore, the gated network output of task *m* is

(8) $$G_{m}=\psi \left(P_{m},O_{m}\right)$$

where ψ is a gated network function, similar to that in the base model. So, the output of the tower layer of task *m* is

(9) $$Y_{m}=t^{m}\left(G_{m}\right)$$

where $t^{m}$ denotes the tower network of task *m*.

### 3.3. Pruning-Based Feature Sharing Strategy

In the proposed model, the parameters required by different tasks are taken into account, and the noise parameters that would cause negative transfer are pruned. The two ideas are based on the following assumptions:

**Hypothesis** **1.** *In MTL, diverse tasks need different representations among shared experts. That is, for any task, the task can find an optimal subnet architecture that is most suitable for itself by pruning the shared expert’s parameters;*

**Hypothesis** **2.** *After the base model is trained, the weight of noise features that will affect the performance of the model will have a lower weight, and when parameters pruning is applied to these connections, there will be no adverse impact within a reasonable threshold.*

For Hypothesis 1 Frankle J. et al. [29] conducted a lot of experiments to prove that in an original base model, a more sparse pruned sub-model can always be found, and this sub-model can reach or even exceed the performance of the original model in roughly the same training process as the original model and the number of iterations. For Hypothesis 2 Sun T et al. [12] warmed up the original model before pruning so that a specific subnet could be confirmed before pruning model parameters related to the task. Then, the cut parameters with IMP according to the neurons’ weight value for obtaining the subnet of the task will hardly be harmful to the model performance in the next training process. Moreover, the pruning mechanism of the neural network can reduce more than 90% of parameters in the trained network, thus cutting down storage requirements and improving computing performance without affecting accuracy [30].

**Figure 3:**
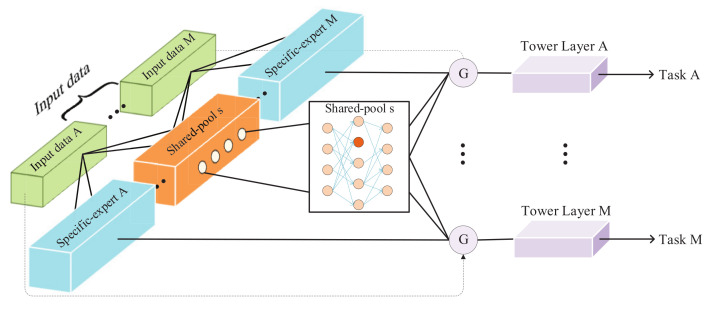
Model architecture of PBFS.

Following the sparse sharing mechanism, our PBFS method provides an effectively shared architecture (shown in Figure 3). Given a base dense neural work, the *M* task and the sparsity parameter of each task to each shared pool are used to generate shared pool *Q*, which has ruled the strategy of the task’s information sharing (described in detail in Algorithm 1). Furthermore, its output representation is routed to the gated network of the corresponding task, and the parameters pruning is performed inside the shared pool. It should be noted that different tasks prune the same shared pool with different target sparsity. Each task has its adjustable pruning parameters for each shared pool, such as initial sparsity, target sparsity, etc., and finally generates an independent training subnet for each task. This subnet contains the parameters required by the corresponding task in the base network. The details are in Algorithm 2.
**Algorithm 1:** Generate sharing pool for tasksInput: *M* tasks, threshold parameter α, correlation matrix ${\mathrm{COOR}}_{M}$Output: Set of sharing pool *Q*Preparation: Generate task pair set *T*={<i,j>,i,j⊆M and i≠j},<i,j>=<j,i>,*S*=0for <i,j> in *T*:      if *Q*==NULL :            *S*=1;            *Q*[1]<—<i, j>;      else:            for s=1 to S+1:                  if $(|SMF_{s}-{\mathrm{COOR}}_{M}^{<i,j>}|<\alpha$ and s<=S):                        *Q*[*s*]<—<*i*, *j*>;                        update $SMF_{s}$;                        break;                  else if (s==S+1):                        S++;                        *Q*[*s*]<—<*i*,*j*>;                        $SMF_{s}$=${\mathrm{COOR}}_{M}^{<i,j>}$;                        break;            end forend forReturn: *Q*

**Algorithm 2:** Generate sparse shared-expert for each task
Preparation: Task *M*, target sparsity $M_{m\to s}^{\lambda }$,(m=1toM, *s* is the shared pool s),
                  initial sparsity $M_{m\to s}^{\mu }$, sharing pool set *Q*
Output: Sparse shared-expert network $\left\{P_{1},P_{2},\ldots ,P_{M}\right\}$
1:for *s* in *Q*:
      generate base shared-expert network $P^{s}\left(x_{s}\right)$,($x_{s}$ is input data of all tasks in Q[s]).
2:for m=1 to *M*:
      for *s* in $U_{m}$:
            $P_{m}^{s}$= prune $M_{m\to s}^{\mu }$ % parameters of $P^{s}$ with low magnitude, save $P_{m}^{s}$ in $P_{m}$.
      end for
end for
And then train model with $P_{m}$.
3:Prune parameters with polynomial decay and training until shared-expert’s sparsity
arrived to $M_{m\to s}^{\lambda }$, record the performance of training process with different sparsity.
4:Replace $P_{m}^{s}$ with the sparse shared-expert reached best performance of task *m*.
5: Repeat steps 3–4 until training is over.
Return: $\left\{P_{1},P_{2},\ldots ,P_{M}\right\}$


After introducing our parameters pruning strategy into the base model, the joint loss optimization of the model can be expressed as

(10) $$L\left[\left(\theta_{1},\theta_{1}^{s}\right),\left(\theta_{2},\theta_{2}^{s}\right)\ldots ,\left(\theta_{M},\theta_{M}^{s}\right)\right]=\sum_{m=1}^{M}w^{m}L_{m}\left(\theta_{m},\theta_{m}^{s}\right)$$

where $\theta_{m}$ denotes the task m’s specific-experts parameters, and $\theta_{m}^{s}$ is task *m*’s shared parameters, and $L_{m}$ indicates the loss function of task *m*.

## 4. Experiments and Results

### 4.1. Datasets

UCI census income [31]: This dataset is extracted from the U.S. Census database and contains 299,285 statistical data instances of adults, each of which has 40 features. This experiment set two classification tasks as income and marital status, respectively. The former predicts whether the income of each sample is higher than 50,000, and the latter classifies married or unmarried. The correlation score of the two tasks is 0.1768 [9].

Movielens 1 M: This dataset is widely used in the field of recommendation systems, including 100,000 rating records (1–5 points) of 1682 films by 943 users, of which each user has no less than 20 rating data for different films. In this dataset, the ratio of training data to test data is 7:3, and the prediction of users’ age and rating to films are regarded as regression and classification task, respectively. When the rating score is greater than 3, the user is deemed to like the movie and otherwise to not like it. The correlation score of the two tasks is 0.0551.

Student [32]: This data approaches student achievement in secondary education of a Portuguese school. The datasets are provided regarding the performance in two distinct subjects: mathematics and Portuguese language. It is worth noting that the target attribute G3 has a strong correlation with attributes G2 and G1. This occurs because G3 is the final year grade, while G1 and G2 correspond to the 1st and 2nd period grades. It is more challenging to predict G3 without G2 and G1, but such prediction is much more useful. So we consider both G1 and G2 as unattached tasks, and G3 cannot learn related information, except the sharing pool that we build. Task’s correlation score of student dataset shown in Table 1.

Synthetic data: This dataset was generated by nonlinear rules in [9] to verify the performance of the model on tasks with different correlated scores, and two regression tasks were generated in total. In the experiment of this paper, we use this rule to generate 120,000 data, of which 100,000 are used for the training model, 10,000 are applied for verification, and the rest for testing.

### 4.2. Comparison Model

Single-task: Two separate MLP networks are applied to predict different tasks, respectively.

Shared-bottom [5]: A widely used method in the multi-task model. Its basic idea is to make different tasks share a knowledge extraction network, and only the output of the tower layer is separated.

Cross-stitch [3]: This model uses two cross-sharing units to learn the common knowledge between two tasks, a coefficient matrix to learn the output of hidden layers of different tasks, and controls the shared knowledge learning between various tasks by automatically adjusting the value of parameters in the coefficient matrix.

MMOE [9]: MMOE proposed to use a gated unit that relied on input samples to control the importance of each expert for each task so that different tasks have a specific selection ability on the same expert.

PLE [13]: This model separates task-specific experts and shared experts, whose information is selectively learned by a gated network.

### 4.3. Experiments Setup

For all the comparison algorithms, we adjust parameter settings based on their models to ensure that the learning process is neither under-fitting nor over-fitting to achieve the best performance of the corresponding model on each dataset. Since the tunable parameters of each model are different, we refer to the experimental parameter design in the original paper of the comparison algorithm and summarize the detailed data of parameters after the experiment as follows:

Single-task: Experiments on all datasets use a three-layers MLP network with hidden layer size of [32,16,8], and the number of neurons in the final output layer is determined by task attributes.

Shared-bottom: The shared-bottom mechanism uses the same three-layer MLP network as the single-task model, but the difference is that it has a task-specific tower layer, which is a two-layer dense network with an input layer and an output layer. The number of neurons in the input layer is eight, and the task attributes also decide the output layer size.

Cross-stitch: According to the cross-stitch model’s design, 32 neurons are used in both the sharing and task layers, and the output layer is set as above.

MMOE: This model adopts the experimental setup in the original paper [9], with a total of eight task-specific experts and four neurons for each expert.

PLE: Two layers of experts are set according to the original paper. Each expert is a single-layer network containing 16 neurons.

PBFS: Each expert has 2 hidden layers in the census-income dataset, and each layer size has 32. In the MovieLens dataset, each layer has 16 hidden units. In the synthetic dataset, eight neurons are set up in PBFS.

The Adam optimizer is used to learn all models, the initial learning rate on the census income dataset is 0.001, and the number of iterations is set to 400. The learning rate on the MovieLens and synthetic datasets are both 0.0001, and the former training has 400 epochs, yet later has 300 epochs. Furthermore, the input data in the multi-task models are executed simultaneously, and the shared subnets of different tasks in the proposed model are trained in parallel.

There are only two tasks in the census income, MovieLens, and synthetic datasets for the proposed model, so there is no need to design the number of shared-pool and shared-threshold parameter α. However, there are three tasks in the student dataset, so in addition to the primary parameter design of the unit’s number, the design of other parameters will be discussed in the tuning of hyperparameters. The cross-entropy loss function is used for classification tasks, and the MSE loss function is introduced to regression tasks on all datasets.

### 4.4. Experiments Results

#### 4.4.1. Results in Public Datasets

Both tasks in the census-income dataset are classification tasks. Therefore, the F1-score and ACC are selected to evaluate the proposed model. Table 2 describes the performance of different multi-task models on this dataset, and the optimality is marked in bold black font in Table 2.

The single-task model has the highest ACC value on task 1, due to only needing to improve the prediction accuracy of one target, compared to the multi-task model aiming to improve the performance of multiple targets simultaneously. From the perspective of parameter complexity and feature learning, the single-task model is simpler and more efficient in the learning process. However, when the tasks are correlated, the single-task model would damage the final effect because it does not learn the common knowledge of other tasks. We can find that the performance of the single-task in the marital task is not better than that of other multi-task models. When the sparsity of the PBFS model is close to 1, it is equivalent to a single-task model; the influence of sparsity on the results is analyzed in detail in the next section.

In addition, we can notice that the performance of the proposed model on task 1 is not significantly improved, compared with other models. In contrast, it is dramatically enhanced on task 2. Inspired by the seesaw phenomenon presented in PLE [13], after analyzing the result of the comparison algorithm on the dataset, we find that when most models (such as the comparison algorithms) improve the prediction performance of one task, they often lead to the performance degradation of another task. This phenomenon also means that when looking for the maximum hidden layer representation fusion of different tasks, the traditional multi-task model favors one task rather than treating two tasks equally. However, the proposed model can significantly improve the martial results without compromising the prediction performance of income because each task has different pruning strategies for the same shared pool. Each task can decide whether to use a hidden feature to generate a mutually independent subnet with some identical parameters and then train the subnet separately.

Table 3 shows the shared pool sparsity of the task subnet when the F1-score and accuracy of each task reaches the optimal value. For the income task, the sparsity of the highest F1 value is different from that when the ACC value is optimal. After comparing the experimental data, we find that when the F1 value is optimal, the difference between the ACC value and the optimal ACC value under the same sparsity is only 0.001; the same error also exists in the F1 value under the optimal sparsity of ACC.

The two tasks in the Movielens dataset are the classification task and regression task, respectively. We use AUC to measure for the classification task, while for the regression task, we use MSE to evaluate. The correlation score between rating and age is deficient, and it is very complex to learn the features that can fit these two tasks by DNN, so many multi-task models struggle to make noticeable progress. Despite this, the performance of PBFS on this dataset is better than that of other multi-task models (shown in Table 4).

For different tasks, different subnet sparsity can be selected according to the experimental results so that the model can trade off multiple tasks and optimize the performance as much as possible. According to the experimental results on the Movielens dataset and Table 5, the sparsity of the shared pool required by task 1 is very different from that needed by task 2. After analyzing the data samples, we observed that the rating data are closely related to the user’s age, but the user’s age is not associated with the rating. That is why the age subtask needs to cut more parameters. However, according to the the Pearson correlation coefficient, the calculated correlation of the two tasks is very low for only considering the linear correlation of the two tasks, which is insufficient to measure the relationship between variables when a particular progressive relationship exists between tasks. That is why the traditional multi-task model is always harmfully affected by the seesaw phenomenon.

There are two subjects in the student dataset, and each subject has three scores, G1, G2 and G3, where G3 is the most crucial task, which indicates students’ final score, yet G1 and G2 exist as auxiliary tasks of G3. Unlike the above experiments, most of the compared models are ruled out because their design is unavailable for three tasks, so we compared PBFS with MMOE and shared-bottom model on the student dataset.

Although the performance of the proposed model on G1 and G2 is not always optimal, it can always maintain the optimal value on G3 (shown in Table 6). In MTL researchAs we all know, selecting appropriate auxiliary tasks for the main task is also a way to optimize the structuremulti-task learning model. The experimental results on the student dataset manifestedfully prove that the proposed model can improve the performance of the main task by uniting subtasks.

#### 4.4.2. Result in Synthetic Dataset

Experiments to discuss the impact of different task correlations are conducted in a synthetic dataset to convincingly demonstrate PBFS’s manifestation in low task relatedness. Model sparsity is also considered for validating adaptive parameters selection capability. Aside from 100,000 samples being generated for the training model in every task correlation score, 10,000 samples are used for validation and test, respectively. Tasked relatedness is governed in [0.2, 0.5, 0.8, 1].

**Figure 4:**
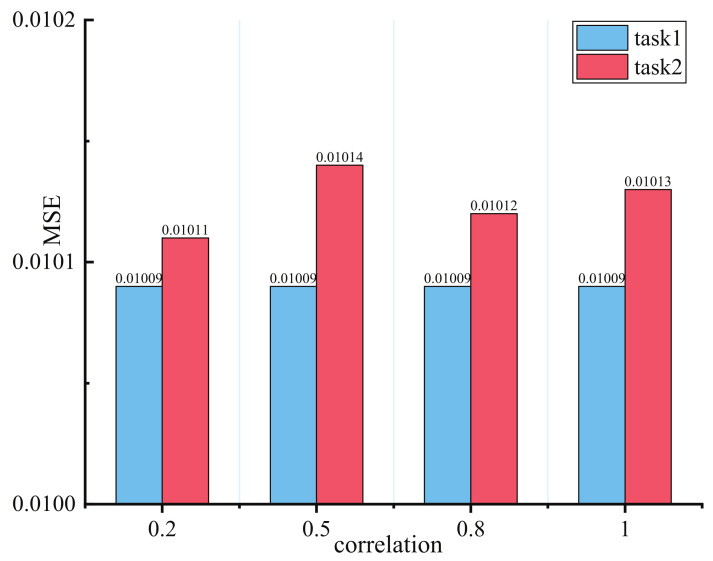
Results with different task’s correlation score on synthetic dataset.

Figure 4 showsshow the lowest MSE value of each task in four distinct task correlation scores, and the performance of the proposed model remains stable beyond expectation as the task correlation decreases. In particular, changes in task relevance have no harmful impact in task 1; although the MSE value in task 2 achieved a slight increase when task correlation score faced 0.5, it also reduced to the lowest value of 0.2 in the correlation score. It is worth noting that this experiment is taken up with different model sparsity, which means that the selected lowest MSE value of two tasks comes from dissimilar trained task subnets. We discuss the performance of task relatedness under unequal subnet sparsity in the next section.

## 5. Analysis and Discussions

### 5.1. Hyperparameters Tuning

In this section, we experiment on the census-income dataset to study the influences of the expert units in PBFS. In detail, the shared expert’s initial sparsity is set to 0.6, the target sparsity is 0.7, and the expert’s units are designed within [8, 6, 32, 64, 128]; BOlded numbers in Table 7 shows the optimal results.

With the expert with 64 units, the proposed model shows outstanding performance in two tasks, but it is not the experimental setting we selected for PBFS on the census-income dataset. To explain the reason, we also discuss the performance differences between 32 units and 64 units with different sparsity. The experimental results are as shown in Table 8.

Notwithstanding that the model achieved the best performance with the task experts with 64 units, the number of parameters with 64 units (including trainable and untrainable parameters) are almost three times that of 32 units, and the former’s performance improvement is hardly noteworthy. Meanwhile, the original intention of the proposed model is to reduce the model parameters as much as possible but keep the model performance excellent. Of course, if high performance is expected in practical application, a scheme with more parameters can also be used.

### 5.2. Subnet’s Sparsity

The proposed model prunes parameters in the shared expert, so we conduct many experiments in all datasets to find the best subnet for each task, and their final shared-expert sparsity is decided by the test results. Therefore, we control the subnet sparsity from 0 to 1. Every experiment is conducted with 0.1 difference in the initial sparsity and the target sparsity, such as finding the best subnet in sparsity from 0 to 0.1, and then recording it.

**Figure 5:**
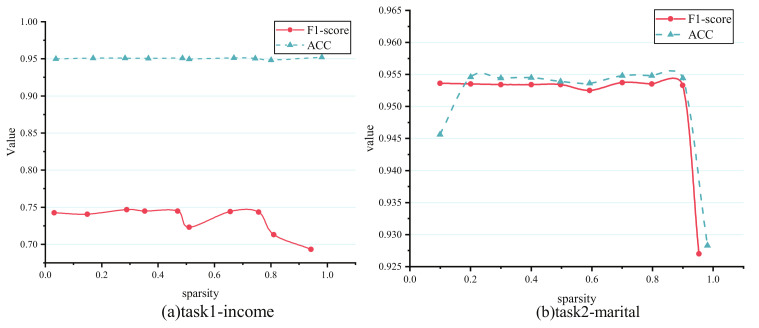
Results of different sparsity on census-income dataset. (**a**) Task 1-income’s performance with subnet sparsity from 0 to 1. (**b**) Task 2-marital’s performance with subnet sparsity from 0 to 1.

Figure 5 shows how different shared-expert sparsity impacts the task prediction results in the census-income dataset, and all tasks’ F1-score or ACC encounter a decline, especially for task 2-marital. Admittedly, PBFS is similar to the single-task model when the sparsity of the shared experts is close to 1, and most equal to CGC when close to 0. Task 2-marital’s sudden dropped accuracy when 100% parameters of the shared experts is nearly pruned show an almost similar performance with the single-task model illustrated in Table 2. Fortunately, the case mentioned above does not happen in task 1-income, whose ACC can remain relatively flat for most subnet sparsity. By comparison, the dependence of task 2 on task 1 is greater than that of task 1 on task 2, which is challenging to find with prior knowledge of researchers; even more than that, the correlation of tasks is measured with an ambiguous value that hinders them from building an effective multi-task learning architecture. In this situation, sharing parameters through task-based pruning is a method that does not cause conflict. The above results indicate that the proposed model could search the most optimal shared-expert subnet as possible for each task, which can match the original shared-experts performance, or even outperform that.

**Figure 6:**
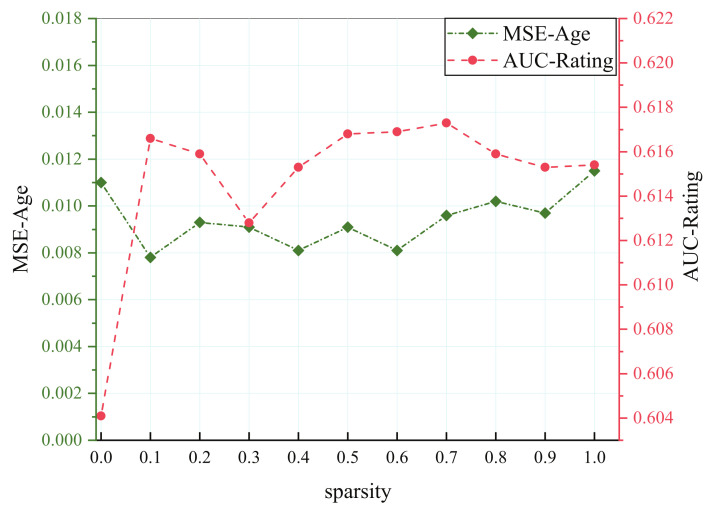
Results of different sparsity on MovieLens dataset.

It is easier to find a hidden representation that can meet multiple closely correlated tasks, while that will result in adverse consequences for aggravating the conflict of parameters in less related tasks. Therefore, most multi-task models work with the former well, yet negative transfer will occur in the latter. Two tasks in the MovieLens dataset are extremely loosely correlated, although the fluctuation continues at different sparsity. The proposed model outstrips the single-task model after trimming several parameters shown in Figure 6.

**Figure 7:**
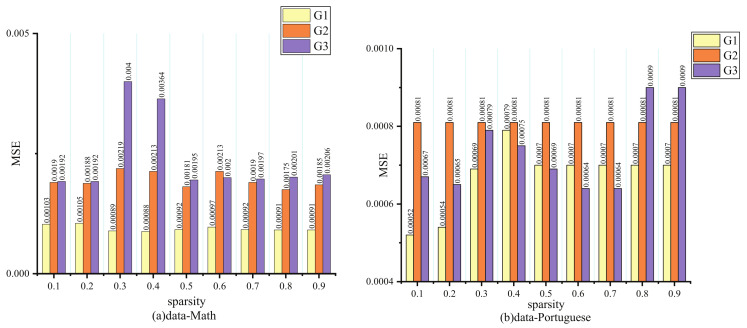
Results of different sparsity on student dataset.

Three tasks in the student dataset have complicated relations when measured with the Pearson correlation coefficient, and G3 has a strong correlation with targets G2 and G1. We can note that G2 always maintains an upper MSE, whatever the sparsity in Figure 7. Actually, G1 and G2 have a low relationship with G3, so their performance is not affected evidently when the sparsity changes. However, for G3, which is closely correlated with other tasks, its performance will be greatly hurt because it inadequately learns the common knowledge when too many parameters are pruned.

**Figure 8:**
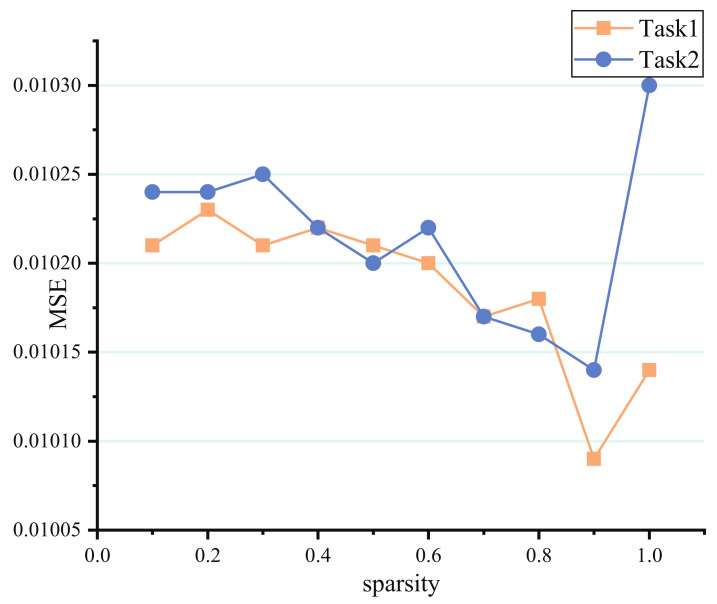
Results of different sparsity on synthetic dataset.

In the same way, PBFS’s MSE value in two tasks of synthetic data is verified with different sparsity under the 0.5 correlation score. According to Figure 8, the task’s MSE reaches the lowest value when the pruning rate is 0.9, but it will rebound obviously when all parameters are cut off, which proved that there might be too many redundant parameters existing in the model. Additionally, the importance of sharing some public information between tasks also provides evidence that the proposed model can improve the prediction accuracy.

### 5.3. Adjustable Parameter α

Parameter α affects the model’s performance by controlling the number of shared pools. Theoretically, it can play a better role among tasks with low correlation. To verify the influence of α on the model performance, we conducted a comparative test on the student dataset, adjusted parameter α according to the relevancy of tasks in the dataset, and obtained a model with three shared pools. Table 9 describes the experimental results. Not surprisingly, because of the high correlation score, the approach of a multi sharing pool is no better than using a single shared expert. Specifically, the former performed better on G1 and G2 but significantly worse on G3. In future work, a dataset with multiple tasks and low correlation between them is expected to verify the effect of parameter α.

## 6. Conclusions

We propose a novel multi-task learning model (PBFS) composed of soft sharing and parameter pruning, introducing a more sparse and efficient structure into the traditional multi-task model. Its distinctive advantages are testified with a lot of experiments on synthetic data and several public datasets, including outperforming other baseline MTL models and alleviating the harmful impact of negative transfer. Furthermore, it can better address the issue, where tasks are less related and search the best shared-experts subnet for each task.

In future work, we will concentrate on introducing information sharing into multi-modal data in NLP, reducing the computational overhead of the whole MTL structure and optimizing the parameter pruning strategy simultaneously.

## Figures and Tables

**Table 1:** Task’s correlation score in student dataset.

	Math			Portuguese		
	G1	G2	G3	G1	G2	G3
G1	1.0000	0.8521	0.8015	1.0000	0.8650	0.8263
G2	0.8521	1.0000	0.9049	0.8650	1.0000	0.9185
G3	0.8015	0.9049	1.0000	0.8263	0.9185	1.0000

**Table 2:** Results on census-income dataset.

Model	Task 1-Income		Task 2-Marital	
	F1-Score	ACC	F1-Score	ACC
Single-task	0.6931	**0.9520**	0.9270	0.9283
Shared-bottom	0.6436	0.8451	0.9313	0.9327
Cross-stitch	0.7423	0.9505	0.9334	0.9345
MMOE	0.6790	0.9482	0.9325	0.9336
PLE	0.7139	0.9509	0.9272	0.9290
PBFS(ours)	**0.7466**	0.9511	**0.9537**	**0.9548**

**Table 3:** Optimal subnet’s sparsity of tasks on census-income dataset.

	Task 1-Income		Task 2-Marital	
	F1-score	ACC	F1-score	ACC
sparsity	0.2891	0.6691	0.7002	0.7002

**Table 4:** Result on MovieLens dataset.

Model	AUC-Rating	MSE-Age
Single-task	0.6154	0.0115
Shared-bottom	0.5387	0.0133
Cross-stitch	0.5977	0.0117
MMOE	0.5836	0.0110
PLE	0.6046	0.0110
PBFS(ours)	**0.6173**	**0.0078**

**Table 5:** Optimal subnet’s sparsity of tasks on MovieLens dataset.

	Task 1-Rating	Task 2-Age
sparsity	0.1016	0.6992

**Table 6:** Result of Portuguese on Student dataset.

Subject	Portuguese			Math		
**Model**	**MSE-G1**	**MSE-G2**	**MSE-G3**	**MSE-G1**	**MSE-G2**	**MSE-G3**
Shared-bottom	**0.0005**	0.0008	0.0009	0.0010	**0.0014**	0.0021
MMOE	0.0008	**0.0006**	0.0007	0.0015	0.0020	0.0053
PBFS	**0.0005**	0.0007	**0.0006**	**0.0008**	0.0017	**0.0019**

**Table 7:** Results on different units.

Units	8	16	32	64	128
Income-F1	0.7179	0.7331	**0.7441**	**0.7448**	0.7138
Marital-F1	0.9457	0.9456	**0.9537**	**0.9550**	0.9536

**Table 8:** Results on different sparsity.

Sparsity		0.1	0.2	0.3	0.4	0.5	0.6	0.7	0.8	0.9	Number of Parameters
32 units	F1-income	0.7425	0.7405	**0.7466**	0.7448	0.7448	0.7228	0.7441	0.7433	0.7128	18,682
	F1-marital	0.9536	0.9535	0.9534	0.9534	0.9534	0.9525	**0.9537**	0.9535	0.9533	
64 units	F1-income	0.7415	0.7413	0.7400	0.7448	0.7439	0.7391	0.7448	**0.7475**	0.7452	61,402
	F1-marital	0.9541	0.9528	0.9545	0.9539	0.9537	0.9548	**0.9550**	0.9538	0.9548	

**Table 9:** Optimal subnet’s sparsity of tasks on MovieLens dataset.

Number of Sharing Pool	Portuguese			Math		
	MSE-G1	MSE-G2	MSE-G3	MSE-G1	MSE-G2	MSE-G3
1	0.0005	0.0007	0.0006	0.0008	0.0017	0.0019
3	0.0005	0.0006	0.0009	0.0007	0.0019	0.0045

## Abbreviations

The following abbreviations are used in this manuscript:

MTL	Multi-task Learning
NLP	Natural Language Processing
DNN	Deep Neural Networks
RNN	Recurrent Neural Networks
LSTM	Long Short-Term Memory
NAS	Neural Architecture Search
AutoML	Automated Machine Learning
UCI	University of California
MLP	Multi Layer Perceptron
MSE	Mean Squared Error
AUC	Area Under the Curve

## References

[1] Ma X., Zhao L., Huang G., Wang Z., Hu Z., Zhu X., Gai K. Entire space multi-task model: An effective approach for estimating post-click conversion rate. Proceedings of the 41st International ACM SIGIR Conference on Research & Development in Information Retrieval.

[2] Hadash G., Shalom O.S., Osadchy R. Rank and rate: Multi-task learning for recommender systems. Proceedings of the 12th ACM Conference on Recommender Systems.

[3] Misra I., Shrivastava A., Gupta A., Hebert M. Cross-stitch networks for multi-task learning. Proceedings of the IEEE Conference on Computer Vision and Pattern Recognition.

[4] Negrinho R., Gordon G. (2017). Deeparchitect: Automatically designing and training deep architectures. arXiv.

[5] Caruana R. (1997). Multitask learning. Mach. Learn..

[6] Collobert R., Weston J. A unified architecture for natural language processing: Deep neural networks with multitask learning. Proceedings of the 25th International Conference on Machine Learning.

[7] Subramanian S., Trischler A., Bengio Y., Pal C.J. (2018). Learning general purpose distributed sentence representations via large scale multi-task learning. arXiv.

[8] Liu X., He P., Chen W., Gao J. (2019). Multi-task deep neural networks for natural language understanding. arXiv.

[9] Ma J., Zhao Z., Yi X., Chen J., Hong L., Chi E.H. Modeling task relationships in multi-task learning with multi-gate mixture-of-experts. Proceedings of the 24th ACM SIGKDD International Conference on Knowledge Discovery & Data Mining.

[10] Søgaard A., Goldberg Y. Deep multi-task learning with low level tasks supervised at lower layers. Proceedings of the 54th Annual Meeting of the Association for Computational Linguistics (Volume 2: Short Papers).

[11] Hashimoto K., Xiong C., Tsuruoka Y., Socher R. (2016). A joint many-task model: Growing a neural network for multiple nlp tasks. arXiv.

[12] Sun T., Shao Y., Li X., Liu P., Yan H., Qiu X., Huang X. Learning sparse sharing architectures for multiple tasks. Proceedings of the AAAI Conference on Artificial Intelligence.

[13] Tang H., Liu J., Zhao M., Gong X. Progressive layered extraction (ple): A novel multi-task learning (mtl) model for personalized recommendations. Proceedings of the Fourteenth ACM Conference on Recommender Systems.

[14] Xiao X., Chen H., Liu Y., Yao X., Liu P., Fan C., Ji N., Jiang X. (2020). LT4REC: A Lottery Ticket Hypothesis Based Multi-task Practice for Video Recommendation System. arXiv.

[15] Qin Z., Cheng Y., Zhao Z., Chen Z., Metzler D., Qin J. Multitask mixture of sequential experts for user activity streams. Proceedings of the 26th ACM SIGKDD International Conference on Knowledge Discovery & Data Mining.

[16] Clarkson G., Jacobsen T.E., Batcheller A.L. (2007). Information asymmetry and information sharing. Gov. Inf. Q..

[17] Ruder S., Bingel J., Augenstein I., Søgaard A. (2017). Sluice networks: Learning what to share between loosely related tasks. arXiv.

[18] Jacobs R.A., Jordan M.I., Nowlan S.J., Hinton G.E. (1991). Adaptive mixtures of local experts. Neural Comput..

[19] Zhao J., Du B., Sun L., Zhuang F., Lv W., Xiong H. Multiple relational attention network for multi-task learning. Proceedings of the 25th ACM SIGKDD International Conference on Knowledge Discovery & Data Mining.

[20] Liu C., Zoph B., Neumann M., Shlens J., Hua W., Li L.J., Fei-Fei L., Yuille A., Huang J., Murphy K. Progressive neural architecture search. Proceedings of the European Conference on Computer Vision (ECCV).

[21] Vanhoucke V., Senior A., Mao M.Z. (2011). Improving the Speed of Neural Networks on CPUs. In Proceedings of the Deep Learning and Unsupervised Feature NIPS Workshop. http://research.google.com/pubs/archive/37631.pdf.

[22] Jacob B., Kligys S., Chen B., Zhu M., Tang M., Howard A., Adam H., Kalenichenko D. Quantization and training of neural networks for efficient integer-arithmetic-only inference. Proceedings of the IEEE Conference on Computer Vision and Pattern Recognition.

[23] LeCun Y., Denker J., Solla S. (1989). Optimal brain damage. Adv. Neural Inf. Process. Syst..

[24] Hassibi B., Stork D.G. (1993). Second Order Derivatives for Network Pruning: Optimal Brain Surgeon.

[25] Li H., Kadav A., Durdanovic I., Samet H., Graf H.P. (2016). Pruning filters for efficient convnets. arXiv.

[26] Liu H., Simonyan K., Vinyals O., Fernando C., Kavukcuoglu K. (2017). Hierarchical representations for efficient architecture search. arXiv.

[27] Elsken T., Metzen J.H., Hutter F. (2018). Efficient multi-objective neural architecture search via lamarckian evolution. arXiv.

[28] Ma J., Zhao Z., Chen J., Li A., Hong L., Chi E.H. Snr: Sub-network routing for flexible parameter sharing in multi-task learning. Proceedings of the AAAI Conference on Artificial Intelligence.

[29] Frankle J., Carbin M. (2018). The lottery ticket hypothesis: Finding sparse, trainable neural networks. arXiv.

[30] Liu Z., Li J., Shen Z., Huang G., Yan S., Zhang C. Learning efficient convolutional networks through network slimming. Proceedings of the IEEE International Conference on Computer Vision.

[31] Asuncion A., Newman D. (2007). UCI Machine Learning Repository. http://archive.ics.uci.edu/ml.

[32] Cortez P., Silva A.M.G. (2008). Using Data Mining to Predict Secondary School Student Performance. http://archive.ics.uci.edu/ml/datasets/Student+Performance.

